# Optimizing Thyroid Nodule Management With Artificial Intelligence: Multicenter Retrospective Study on Reducing Unnecessary Fine Needle Aspirations

**DOI:** 10.2196/71740

**Published:** 2025-07-30

**Authors:** Jia-Hui Ni, Yun-Yun Liu, Chao Chen, Yi-Lei Shi, Xing Zhao, Xiao-Long Li, Bei-Bei Ye, Jing-Liang Hu, Li-Chao Mou, Li-Ping Sun, Hui-Jun Fu, Xiao-Xiang Zhu, Yi-Feng Zhang, Lehang Guo, Hui-Xiong Xu

**Affiliations:** 1 Department of Medical Ultrasound, Shanghai Tenth People's Hospital, Tongji University School of Medicine, YanChang Middle Street 301, Shanghai, China, 86 21-66307539; 2 Department of Thyroid Surgery, Sichuan Provincial People's Hospital, University of Electronic Science and Technology of China, Chengdu, China; 3 Med AI Technology (Wuxi) Co, Ltd, Wuxi, China; 4 Department of Ultrasound, Zhongshan hospital, Institute of Ultrasound in Medicine and Engineering, Fudan University, Shanghai, China; 5 Department of Pathology, Shanghai Tenth People’s Hospital, Shanghai, China; 6 Chair of Data Science in Earth Observation, Technical University of Munich, Munich, Germany; 7 Department of Ultrasound, Shanghai General Hospital, Shanghai Jiao Tong University School of Medicine, Shanghai, China

**Keywords:** thyroid nodules, artificial intelligence, fine needle aspiration, risk stratification, ultrasound

## Abstract

**Background:**

Most artificial intelligence (AI) models for thyroid nodules are designed to screen for malignancy to guide further interventions; however, these models have not yet been fully implemented in clinical practice.

**Objective:**

This study aimed to evaluate AI in real clinical settings for identifying potentially benign thyroid nodules initially deemed to be at risk for malignancy by radiologists, reducing unnecessary fine needle aspiration (FNA) and optimizing management.

**Methods:**

We retrospectively collected a validation cohort of thyroid nodules that had undergone FNA. These nodules were initially assessed as “suspicious for malignancy” by radiologists based on ultrasound features, following standard clinical practice, which prompted further FNA procedures. Ultrasound images of these nodules were re-evaluated using a deep learning–based AI system, and its diagnostic performance was assessed in terms of correct identification of benign nodules and error identification of malignant nodules. Performance metrics such as sensitivity, specificity, and the area under the receiver operating characteristic curve were calculated. In addition, a separate comparison cohort was retrospectively assembled to compare the AI system’s ability to correctly identify benign thyroid nodules with that of radiologists.

**Results:**

The validation cohort comprised 4572 thyroid nodules (benign: n=3134, 68.5%; malignant: n=1438, 31.5%). AI correctly identified 2719 (86.8% among benign nodules) and reduced unnecessary FNAs from 68.5% (3134/4572) to 9.1% (415/4572). However, 123 malignant nodules (8.6% of malignant cases) were mistakenly identified as benign, with the majority of these being of low or intermediate suspicion. In the comparison cohort, AI successfully identified 81.4% (96/118) of benign nodules. It outperformed junior and senior radiologists, who identified only 40% and 55%, respectively. The area under the curve (AUC) for the AI model was 0.88 (95% CI 0.85‐0.91), demonstrating a superior AUC compared with that of the junior radiologists (AUC=0.43, 95% CI 0.36‐0.50; *P*=.002) and senior radiologists (AUC=0.63, 95% CI 0.55‐0.70; *P*=.003).

**Conclusions:**

Compared with radiologists, AI can better serve as a “goalkeeper” in reducing unnecessary FNAs by identifying benign nodules that are initially assessed as malignant by radiologists. However, active surveillance is still necessary for all these nodules since a very small number of low-aggressiveness malignant nodules may be mistakenly identified.

## Introduction

The prevalence of thyroid nodules has been consistently high, with a correspondingly high detection rate [1-3]. Notably, only a tiny proportion of these nodules are malignant [4].

In the real world, fine needle aspiration (FNA) has long been the favored diagnostic method in the management of suspicious thyroid nodules [5]. Before that, different regions used various Thyroid Imaging, Reporting, and Data System guidelines to screen suspicious thyroid nodules that need to receive further FNA [6,7]. However, the decision process of the Thyroid Imaging, Reporting, and Data System results in a significant number of unnecessary FNAs performed for benign lesions [8].

Although false-positive cases cannot be entirely avoided, a recent retrospective study has revealed a higher-than-expected rate of benign thyroid nodules identified after FNAs, reaching up to 50%, and consequently, some of them received unnecessary lobectomy or thyroidectomy [9]. Since these invasive procedures carry significant risks, complications, economic burdens, and psychological distress for patients [4], a better decision-making process is needed to avoid unnecessary FNA. Recent advancements in the management of thyroid nodules, including the use of the multimodality US model and the incorporation of molecular markers for more accurate risk stratification, have contributed to enhancing the precision of diagnostic decisions [10,11]. However, some limitations, including operator dependence, low concordance, invasive procedures, and high expenses, restrict their widespread application.

 Many recent studies have shown that artificial intelligence (AI) has the potential to improve thyroid cancer detection [12-15]. Through extensive training on large datasets of annotated thyroid nodule ultrasound images, these AI models provide valuable assistance for the differentiation between benign and malignant [16-19]. Under this background, some commercial AI applications have been approved for clinical use.

Nevertheless, due to the indolent nature of thyroid cancer, the problem of overdiagnosis, similar to unnecessary FNA, is becoming a more concerning issue [4]. Most commercial AI applications have been developed to screen for potentially malignant lesions. However, despite their maturity, these AI tools are still challenging to implement in routine clinical practice as replacements in determining the benign or malignant nature of thyroid nodules. Several key barriers hinder their integration into real-world settings, including lack of interpretability in decision-making, potential disruption to clinical workflows, and concerns surrounding regulatory approval and ethical considerations.

To address these challenges, our study used the ITS100 (MedAl Technology) AI system not as a replacement for radiologists, but as a second-opinion tool. In clinical practice, radiologists typically identify potentially malignant nodules and, after obtaining informed consent, proceed with FNA for confirmation. Due to the subjective judgment of radiologists, this workflow inevitably results in some benign nodules undergoing unnecessary FNA.

With the hope of improving the clinical diagnostic workflow to avoid overdiagnosis, we conducted this study to test whether AI could act as a safeguard against overdiagnosis by preventing unnecessary FNAs and to identify the patient populations that may benefit most from its use.

## Methods

### Overview

This study is a retrospective multicenter study. The validation cohort consists of data from Shanghai Tenth People’s Hospital and Sichuan Provincial People’s Hospital (dataset 1), and the comparison cohort consists of data from Shanghai Zhongshan Hospital (dataset 2) retrospectively.

### Ethical Considerations

This study was approved by the institutional ethics committees of the participating centers (approval SHSY-IEC-5.0/22XJS36/P01). Written informed consent, including a statement of FNA’s risks and data collection, was obtained from patients before undergoing FNA. All participant data were de-identified, stored with restricted access, and analyzed anonymously; no financial compensation was provided for participation.

### Study Cohorts

Consecutive ultrasound screenings with clear images, complete clinical information, and pathological results were collected from January 2021 to August 2023 at Shanghai Tenth People’s Hospital, Sichuan Provincial People’s Hospital, and Shanghai Zhongshan Hospital in China. All nodules included in this study were initially assessed as suspicious for malignancy by radiologists based on ultrasound features, following standard clinical practice, which prompted further FNA procedures. A subset of these nodules also received surgical treatment based on clinical assessment and ultrasound evaluation. FNA cytology results were categorized according to the 2017 Bethesda System [20]. Among all eligible cases, a nodule was selected based on the following criteria: if there was only one nodule, it was chosen; if there were multiple nodules with the same malignant risk classification, the one with the largest diameter was selected; if the malignant risk classifications differed, the nodule with the highest malignant risk classification was chosen. The inclusion and exclusion criteria are listed in Textbox 1.

**Figure 1.**
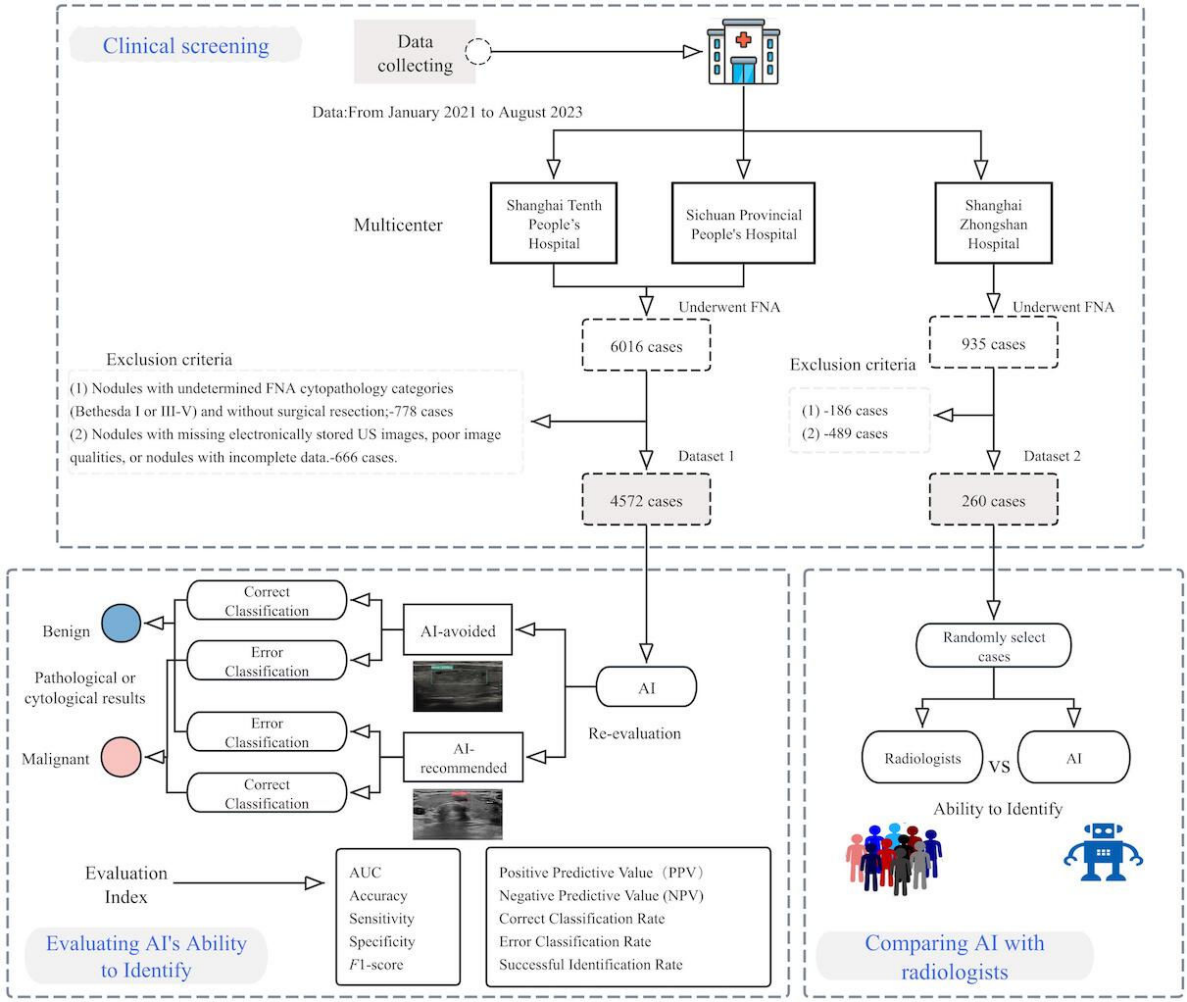
Overview of the study design. AI: artificial intelligence; AUC: area under the curve; FNA: fine needle aspiration.

A detailed flowchart of patient selection is presented in Figure 1.

Textbox 1. **Inclusion and exclusion criteria.**

**Inclusion criteria:**

- Patients aged 18 years or older.
- Nodules that underwent fine needle aspiration (FNA) prompted by radiologists’ suspicion based on ultrasound assessment.
- Participants underwent FNA or surgical procedures to confirm the pathological results, including cytological or histological findings.
- Bethesda II nodules with follow-up data available for more than 12 months.

**Exclusion criteria:**

- Nodules with indeterminate FNA cytopathology categories (Bethesda I or III-V) without pathological results.
- Nodules with missing electronically stored ultrasound images, poor image quality, or incomplete clinical data.

### Grayscale Ultrasound Examination

All nodules underwent ultrasound examinations within 1 week before FNA. The ultrasound examinations were performed using high-frequency linear transducers of real-time ultrasound systems (Multimedia Appendix 1). At least 2 grayscale ultrasound images for each target nodule (the largest transverse and sagittal planes) were routinely recorded.

### Foundation of the AI Algorithm

It is well known that deep learning models require huge amounts of labeled data. To address this challenge in thyroid nodule recognition, an innovative approach has been developed that combines self-supervised learning [21], transfer learning [22], and semisupervised learning [23]. This strategy significantly reduces the demand for labeled data while maintaining high performance.

**Figure 2.**
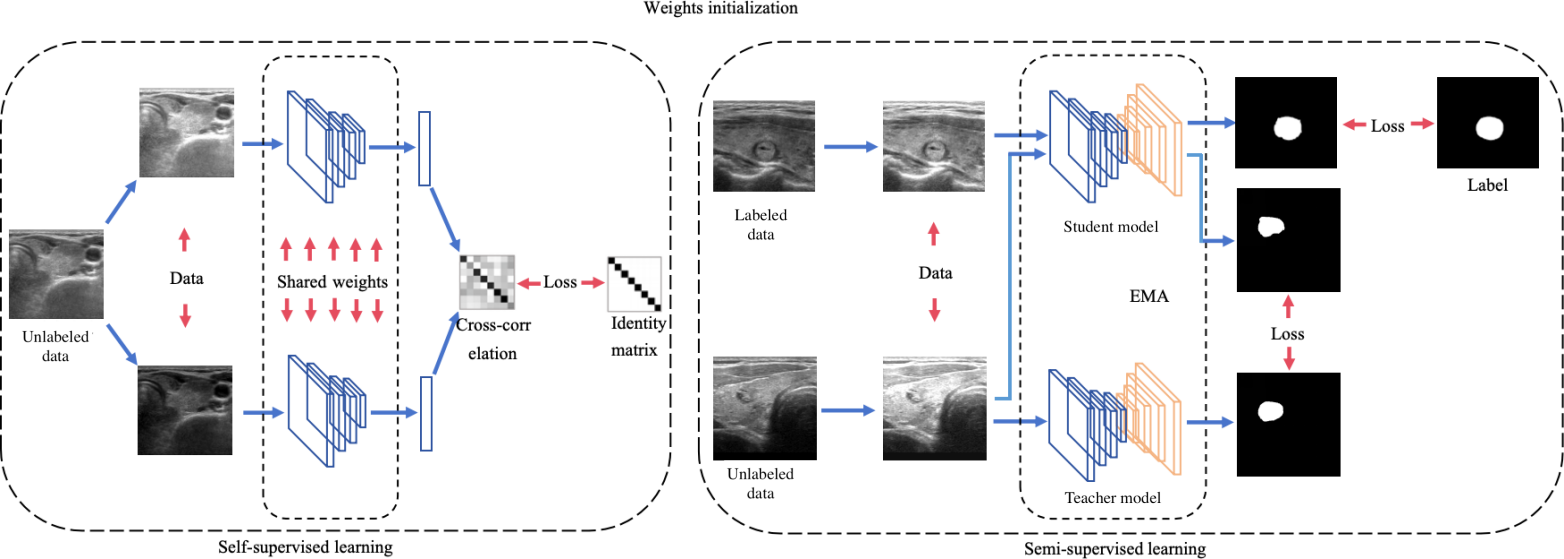
Flowchart of the procedures in the development of artificial intelligence model for thyroid nodule recognition. EMA: exponential moving average.

With the help of the feature extraction capability of convolutional neural networks, the model accurately fits the complex relationship between the content of ultrasound images and the location and nature of thyroid nodules, thus realizing high-precision recognition of thyroid nodules. The flowchart of development is illustrated in Figure 2.

The model development process is divided into 2 phases—pretraining and fine-tuning. In the pretraining phase, a self-supervised learning approach is used to train the model’s encoder network using abundant unlabeled data to learn complex feature representations of ultrasound images. The training strategies of self-supervised learning are inspired by previous studies [21]. The fine-tuning phase introduces a teacher-student model architecture, where both models share the same structure. This phase proceeds as follows: first, initialization—the encoder networks of both the teacher and student models are initialized with the weights obtained from the pretraining phase. In contrast, the decoder networks start with default weight initializations. Second, training—the student model undergoes training using labeled and unlabeled data. For the unlabeled dataset, pseudolabels generated by the teacher model are used. Third, model updates—the teacher model’s parameters are regularly updated using an exponential moving average of the student model’s parameters, ensuring a stable and consistent learning process.

The AI used in this study is called ITS100 and is manufactured by MedAl Technology. The ITS100 system diagnoses thyroid nodules from a real-time ultrasound video, providing “benign” or “malignant” as the label of each nodule. Its core utility has been developed based on state-of-the-art deep learning algorithms, consisting of more than 300,000 images and hundreds of additional videos. Previous studies have demonstrated that this AI has significant value and accuracy in distinguishing between benign and malignant thyroid nodules [24,25].

### Task 1: Evaluating the Ability of AI to Identify Benign Nodules Initially Deemed at Malignant Risk by Radiologists

Nodules deemed to be at risk for malignancy by radiologists in dataset 1 were used to validate the AI. For this AI system, if the AI model classified a nodule as benign, it was hypothesized as “AI-avoided,” and FNA was considered unnecessary. Conversely, if the AI classified a nodule as malignant, it was hypothesized as “AI-recommended” and subsequently underwent FNA. In addition, we defined 3 new metrics to evaluate the AI’s ability to distinguish benign nodules and reduce unnecessary FNA. Furthermore, we define the following metrics to evaluate AI’s abilities:

- Unnecessary FNA rate = $\frac{True\;Benign\;}{Total\;FNA}$

defined as the proportion of FNAs performed on nodules that were ultimately confirmed to be benign.

- Correct classification rate = $\frac{True\;Benign\;AI\;Classification}{\;AI\;Classifications\;as\;Benign}$

defined as the proportion of nodules correctly identified as benign among all nodules classified as benign by the AI.

- Error classification rate = $\frac{True\;Malignant\;AI\;Classification\;as\;Benign}{All\;AI\;Classifications\;as\;Benign}$

defined as the proportion of malignant nodules incorrectly classified as benign among all nodules classified as benign by the AI.

- Successful identification rate = $\frac{True\;Benign\;AI\;Classification}{All\;True\;Benign}$

defined as the proportion of truly benign nodules correctly identified as benign by the AI out of all benign nodules.

### Task 2: Comparison Between Radiologists and AI

Dataset 2 was used to compare the performance of the AI system with that of radiologists in identifying potential benign nodules from those initially deemed at malignant risk by radiologists. A total of 21 radiologists participated in this analysis, including 10 senior and 11 junior practitioners. We randomly selected 30 cross-sectional or longitudinal images of thyroid nodules that had been previously flagged as malignant risk by radiologists in real clinical settings. The radiologists were tasked with identifying nodules that were likely benign and for which FNA could have been avoided, based solely on the imaging data provided, without any external prompts or guidance. This setup aimed to assess their ability to discern unnecessary FNA within a risk cohort.

### Statistical Analysis

In task 1, to evaluate the performance of the AI, the ROC curve was constructed. Sensitivity, specificity, positive predictive value, negative predictive value, accuracy, area under the curve (AUC), and *F*_1_-score were also calculated. The AUC was calculated along with 95% CIs. In task 2, the Delong test was performed to compare radiologists’ and AI’s AUCs. The specific numbers of nodules where the prediction results of the AI were inconsistent with the confirmed pathological results were determined using the confusion matrix. The successful identification rate and correct classification rate were calculated along with 95% CIs. The Mann-Whitney *U* test was used to compare the successful identification rate and correct classification rate between senior radiologists and junior radiologists.

All statistical analyses were conducted using SPSS (Version 22.0, IBM Corporation), R software (Version 4.2.1, R Foundation for Statistical Computing), and Python (version 3.7.13, Python Software Foundation). Results were considered statistically significant at the *P*<.01 level.

## Results

### Characteristics of Data

A total of 6016 cases in dataset 1 and 935 in dataset 2 were recruited. After applying the exclusion criteria, a total of 4572 cases were included in dataset 1 and 260 cases were included in dataset 2, as demonstrated in the workflow in Figure 1. Table 1 illustrates the clinical characteristics of the dataset comprising 4572 patients assessed for malignant risk by radiologists. The cohort predominantly consists of female patients, accounting for 75.3% (3444/4572), with a mean age of 49.4 (SD 13.75) years, reflective of a middle-aged population. The number of lesions in the right lobe was the highest among the cohort. American Thyroid Association (ATA) guidelines categorization revealed 3508 nodules in category 3, a total of 730 nodules in category 4, and 334 nodules in category 5, indicating that most nodules present with low to intermediate suspicion of malignancy. Bethesda categorization yielded the following distribution: category II (n=3106), III (n=137), IV (n=10), V (n=348), and VI (n=971), with a predominance of benign diagnoses (Bethesda category II). The definitive diagnosis classified 3134 nodules as benign and 1438 as malignant.

**Table 1.** Patient demographics and characteristics of thyroid nodules.

Characteristics	Total (age: mean 49.4, SD 13.75 years), n	Benign (age: mean 51.5, SD 13.6 years), n (%)	Malignant (age: 48.5, SD 13.8 years), n (%)
Patients	4572	3134 (68.5)	1438 (31.5)
Patient sex			
Male	1128	713 (22.8)	415 (28.9)
Female	3444	2421 (77.2)	1023 (71.1)
Location			
Right	2387	1635 (52.2)	752 (52.3)
Left	2010	1405 (44.8)	605 (42.1)
Isthmus	175	94 (3)	81 (5.6)
ATA^a^ category			
Low suspicion	3508	2851 (91)	657 (45.7)
Intermediate suspicion	730	202 (6.4)	528 (36.7)
High suspicion	334	81 (2.6)	253 (17.6)
Bethesda category			
II	3106	3078 (98.2)	28 (1.9)
III	137	31 (1)	106 (7.4)
IV	10	7 (0.2)	3 (0.2)
V	348	13 (0.4)	335 (23.3)
VI	971	5 (0.2)	966 (67.2)

a ATA: American Thyroid Association.

### Accuracy Data of AI

**Figure 3.**
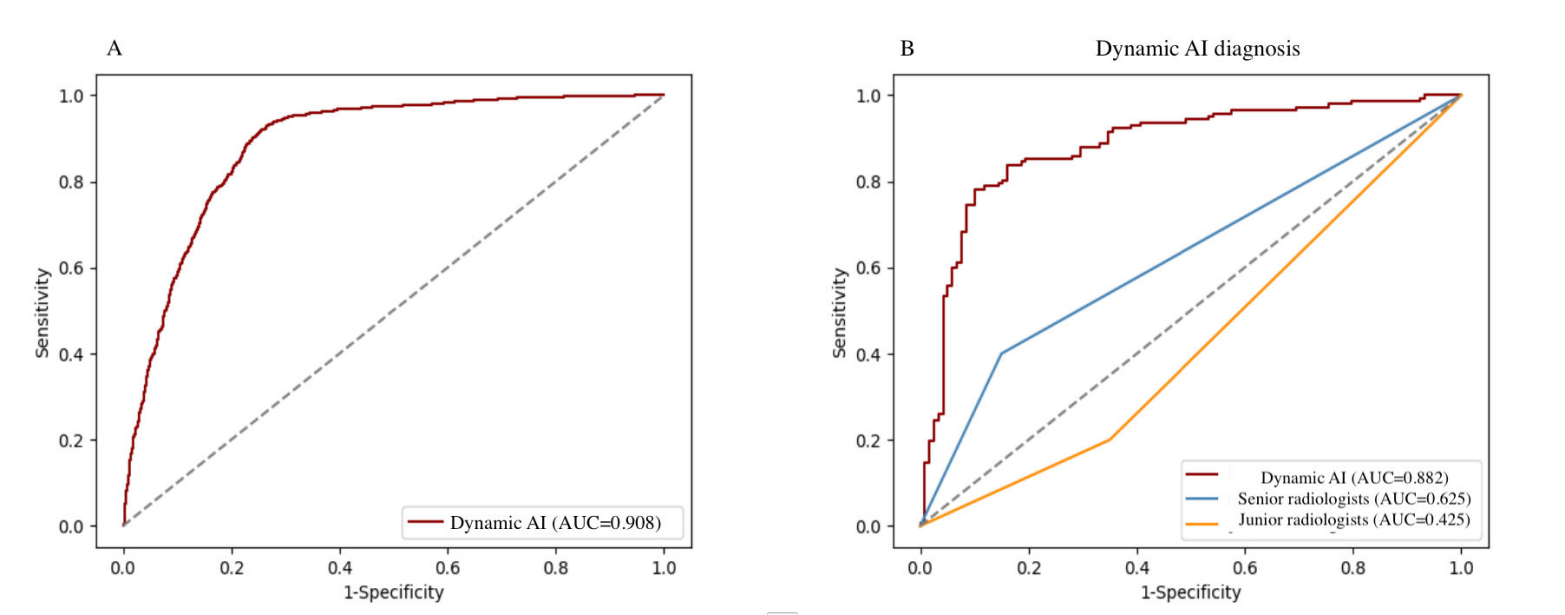
Receiver operating characteristic curves of the artificial intelligence in (**A**) dataset 1 (**B**) and dataset 2. Numbers in parentheses are areas under the receiver operating characteristic curves. Dynamic AI: ITS100 AI system. AI: artificial intelligence; AUC: area under the curve.

We evaluated the performance of the AI in identifying potential benign nodules from those initially classified as at malignant risk by radiologists in dataset 1. The AUC of the AI was 0.91 (95% CI 0.88‐0.93; Figure 3A). The AI’s sensitivity was 91.4% (1315/1438), while its specificity was 86.8% (2719/3134). The positive predictive value was 76% (1315/1730) and the negative predictive value was 95.7% (2719/2842), reflecting a high likelihood that negative test results are accurate. Overall accuracy was 88.2% (4034/4572), demonstrating the AI’s efficacy across 4572 cases. The *F*_1_-score, a harmonized measure of test accuracy, was 0.83, indicating a balanced precision and recall. After applying AI, the number of unnecessary FNAs significantly decreased to 415 (9.1%), compared with 3134 (68.5%) in real-world human readings.

**Figure 4.**
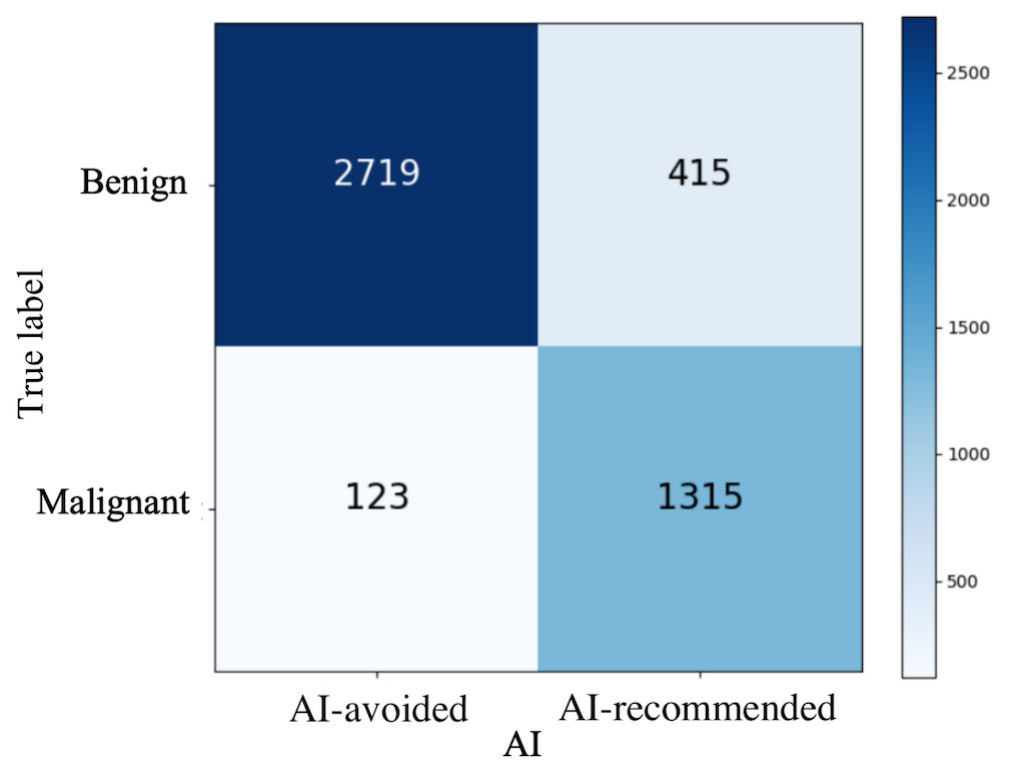
Confusion matrix for artificial intelligence identification results. The y-coordinate is the cytologically (Bethesda II or VI) or pathologically confirmed result. The x-coordinate is the artificial intelligence prediction result. AI: artificial intelligence.

**Figure 5.**
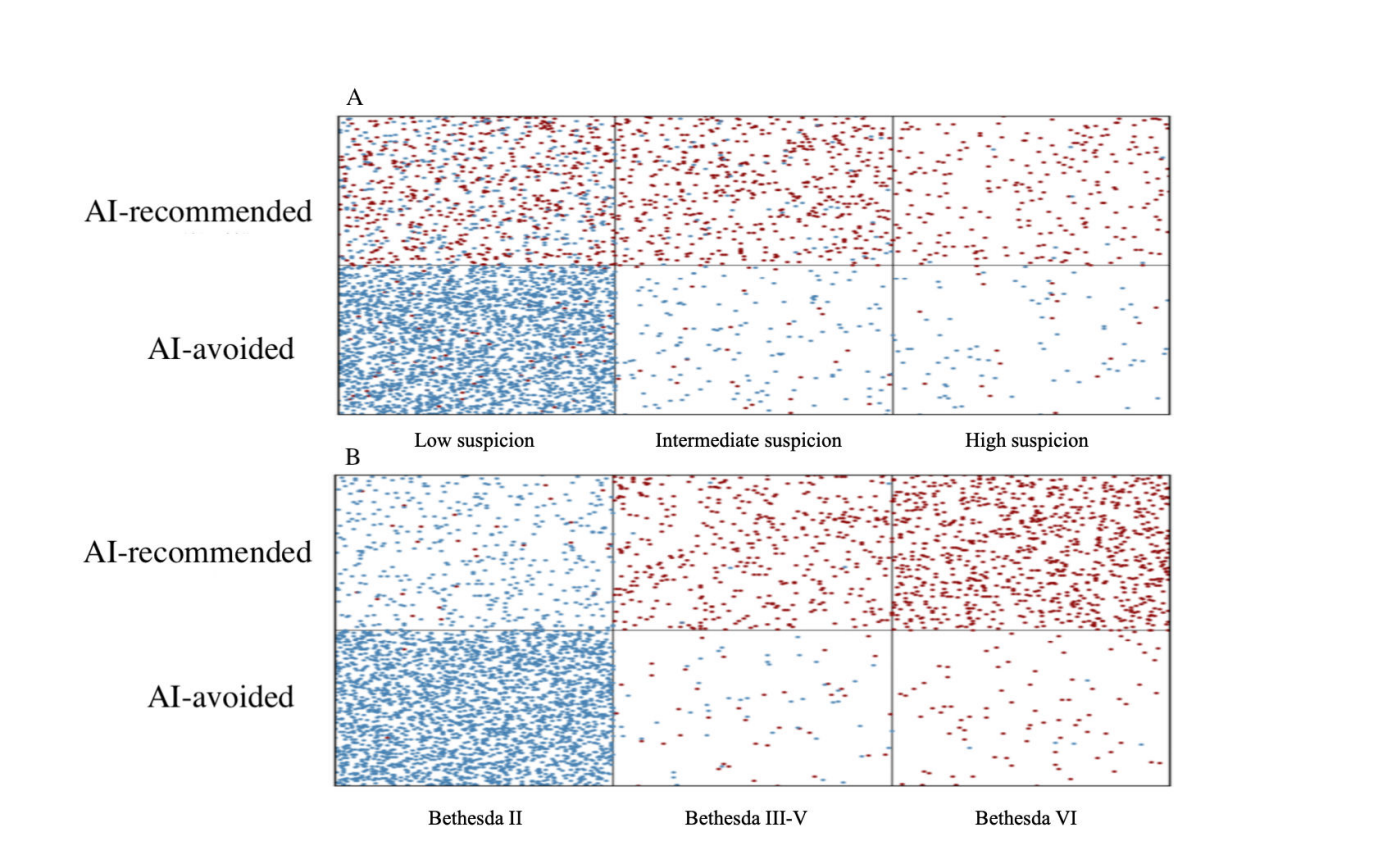
Scatter plot of artificial intelligence binary classification for (**A**) fine needle aspiration across Bethesda categories and (**B**) suspicion categories. Red dots indicate cytologically (Bethesda VI) or pathologically confirmed malignant nodules. Blue dots indicate cytologically (Bethesda II) or pathologically confirmed benign nodules. AI: artificial intelligence.

Figure 4 displays the confusion matrix, showing that the AI correctly identified 2719 benign nodules and 1315 malignant nodules. It also demonstrates a relatively low rate of false negatives. Figure 5A shows the scatter plot comparing the AI’s positive and negative predictions against the Bethesda system’s classifications, which range from benign (Bethesda II) to suspicious for malignancy (Bethesda III-V) to malignant (Bethesda VI). The dots represent individual cases, with red indicating a positive prediction (malignant) and blue indicating a negative prediction (benign). For Bethesda 2, there are many blue dots (AI negative) in the lower left quadrant, suggesting strong agreement on benign diagnoses. For Bethesda 6, the concentration of red dots in the upper right quadrant indicates a high level of agreement on malignant diagnoses. Figure 5B displays a scatter plot of the AI predictions based on the ATA classification, with the “low suspicion” category indicating probably benign nodules, the “intermediate suspicion” category indicating suspicious nodules, and the “high suspicion” category indicating highly suspicious nodules for malignancy. The low suspicion category predominantly shows blue dots, suggesting that the AI largely agrees with the benign diagnosis of these nodules. The intermediate category shows a dense mix, with many cases classified as suspicious by radiologists also being predicted as AI-avoided nodules. The high suspicion category, which should theoretically contain the most malignant nodules, shows a high concentration of red dots, highlighting the AI’s ability to accurately identify highly suspicious cases.

### Rates of Unnecessary FNAs and Other Metrics

The majority of nodules recommended for FNA were classified in Bethesda category VI, accounting for 51.1% (884/1730) of the total, while most of the nodules identified as benign by AI were in Bethesda category II, comprising 94% (2671/2842; Table 2). The unnecessary FNA rates based on human readings for ATA guideline categories were 81.3% for low suspicion, 27.7% for intermediate suspicion, and 24.3% for high suspicion, with an overall unnecessary FNA rate of 68.5% (Table 3).

**Table 2.** Bethesda classification of artificial intelligence–avoided and artificial intelligence–recommended nodules.

Bethesda category	AI^a^-recommended (N=1730), n (%)	AI-avoided (N=2842), n (%)
II	435 (25.1)	2671 (94)
III-V	411 (23.8)	84 (3)
VI	884 (51.1)	87 (3.1)

a AI: artificial intelligence.

**Table 3.** The performance of artificial intelligence across different American Thyroid Association guideline categories.

	Low suspicion	Intermediate suspicion	High suspicion	All
Total	3508 (76.7)	730 (16)	334 (7.3)	4572 (100)
Benign	2851 (81.3)	202 (27.7)	81 (24.3)	3134 (68.5)
Malignant	657 (18.7)	528 (72.3)	253 (75.7)	1438 (31.4)
Unnecessary FNAs^a^	2851 (81.3)	202 (27.7)	81 (24.3)	3134 (68.5)
AI^b^-avoided	2587 (73.7)	165 (22.6)	90 (26.9)	2842 (62.2)
Benign	2510 (97)	137 (83)	72 (80)	2719 (95.7)
Malignant	77 (3)	28 (17)	18 (20)	123 (4.3)
Correct classification rate	2510 (97)	137(83)	72 (80)	2719 (95.7)
Successful identification rate	2510 (88)	137 (67.8)	72 (88.9)	2719 (86.8)
Error classification rate	77 (3)	28 (17)	28 (20)	123 (4.3)

a FNA: fine needle aspiration.

b AI: artificial intelligence.

The AI system demonstrated promising performance across different ATA guideline categories. For low suspicion nodules, the AI avoided 73.7% (2587/3508) of thyroid nodules, with a correct classification rate of 97%. In the intermediate suspicion category, the AI avoided 22.6% (165/730) of thyroid nodules and achieved a correct classification rate of 83%. For high suspicion nodules, the AI avoided 26.9% (90/334), with a correct classification rate of 80%. However, the error classification rates increased with malignancy likelihood, being 3% for low suspicion, 17% for intermediate suspicion, and 20% for high suspicion. The successful identification rates were 88%, 67.8%, and 88.9% for the low, intermediate, and high suspicion categories, respectively (Table 3).

**Figure 6.**
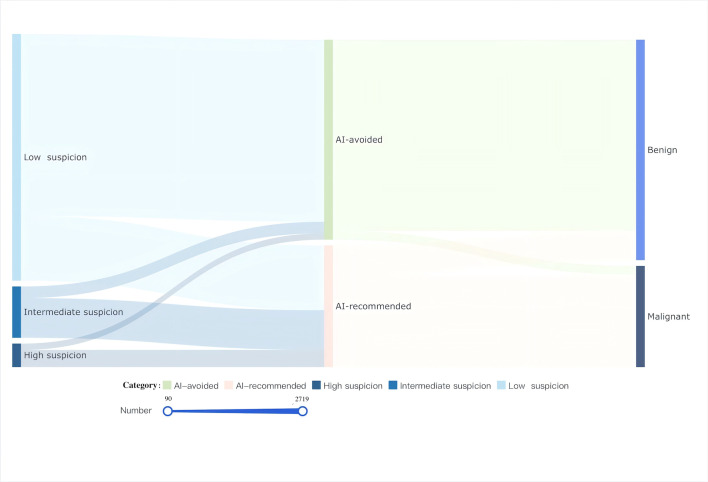
Sankey diagram illustrating the artificial intelligence’s performance across low, intermediate, and high suspicion categories in thyroid nodule evaluation. Links represent the flow between entities. The width of the link corresponds to the amount of flow, with values ranging from 90 to 2719. Colors are used to differentiate between different types of flows. The terms “benign” and “malignant” refer to the results of pathological or cytological tests. AI: artificial intelligence.

The Sankey diagram (Figure 6) highlights that most benign nodules (n=2719, 86.8% of all benign nodules) were successfully avoided by the AI system, with low suspicion nodules comprising 91% (2587/2842) of all AI-avoided nodules. However, 415 benign nodules (9.1% of all nodules) were not identified. Notably, the AI misclassified 123 malignant nodules (2.7% of all nodules) as benign.

Further analysis of the 123 malignant nodules misclassified by AI, as detailed in Table 4, revealed that all were papillary thyroid carcinomas, with 56.9% (70/123) being papillary thyroid microcarcinomas. Among these, 20.3% (25/123) exhibited extrathyroidal extension, 18.7% (23/123) presented with central lymph node metastases, and 74% (91/123) carried the BRAF V600E mutation. Multimedia Appendix 2 shows that the error classification rates of the AI system increased with higher malignancy risk, being lowest for low suspicion nodules (3%) and highest for high suspicion nodules (20%).

**Table 4.** Composition of malignant nodules identified as artificial intelligence–avoided.

Clinical and pathological features	Number, n (%)	
Papillary thyroid microcarcinoma	70 (56.9)	
Other thyroid carcinoma (FTC^a^, MTC^b^, and ATC^c^）	0 (0)	
Extrathyroidal extension	25 (20.3)	
Central lymph node metastasis	23 (18.7)	
Lateral lymph node metastasis	0 (0)	
Distant metastasis	0 (0)	
BRAF V600E mutation	91 (74)	
TERT^d^ promoter mutations	0 (0)	

a FTC: follicular thyroid carcinoma.

b MTC: medullary thyroid carcinoma.

c ATC: anaplastic thyroid carcinoma.

d TERT: telomerase reverse transcriptase.

### Comparison of Radiologists’ and AI Performance

Task 2 compares the performance of radiologists and AI in identifying benign nodules initially assessed as malignant by radiologists, using the correct classification rate and successful identification rate as evaluation metrics. The violin diagram (a) in Multimedia Appendix 3 reveals that junior radiologists exhibited a median successful identification rate of 0.40 (95% CI 0.28‐0.53), while senior radiologists demonstrated a median successful identification rate of 0.55 (95% CI 0.38‐0.71; *P*=.001). AI achieved a successful identification rate of 0.81, surpassing the performance of both junior and senior radiologists. According to the violin diagram (b) in Multimedia Appendix 3, the median correct classification rate for junior radiologists was 0.54 (95% CI 0.44‐0.62), whereas senior radiologists exhibited a median correct classification rate of 0.64 (95% CI 0.48‐0.72; *P*=.13). Furthermore, the datasets indicate that AI achieved a successful identification rate of 0.828, which exceeded the correct classification rate of both junior and senior radiologists (Multimedia Appendix 3).

Figure 3B illustrates the performance of junior and senior radiologists, as well as the AI, in task 2. The AUC values for the AI model were 0.88 (95% CI 0.85‐0.91), demonstrating superior performance to that of junior radiologists with an AUC of 0.43 (95% CI 0.36‐0.49; *P*=.002) and senior radiologists with an AUC of 0.63 (95% CI 0.55‐0.70; *P*=.003).

## Discussion

### Principal Findings

Thyroid nodules, frequently detected during ultrasound, require accurate characterization to assess malignancy and guide patient care. However, the subjective nature of radiological interpretation and the difficulty in distinguishing benign from malignant nodules often lead to unnecessary FNAs. AI holds significant potential in overcoming these challenges by identifying complex patterns and subtle features in ultrasound images that may be missed by human observers [26,27].

In this study, the rate of unnecessary FNAs stood at a high level in dataset 1, as high as the previous study reported [28], which was understandable. The high rate of overdiagnosis aligns with real-world clinical experiences, where various factors, such as human judgment, environmental influences, psychological aspects, and inherent subjectivity in radiological interpretations, play a significant role. Fortunately, based on our findings, after integrating an AI re-evaluation step before performing FNA, the system correctly identified 2719 out of 3134 (86.8%) benign nodules. As a result, the proportion of unnecessary FNAs among all nodules decreased from 3134 out of 4572 (68.5%) to 415 out of 4572 (9.1%).

We found that 2587 out of 3508 (73.7%) low-suspicion nodules based on ultrasound features were identified by AI, with 2510 out of 2587 (97%) correctly classified. In contrast, only 90 out of 334 (26.9%) high-suspicion nodules were identified, with 72 out of 90 (80%) correctly classified. These results suggest that AI re-evaluation is particularly beneficial for low-suspicion nodules and could be routinely implemented before FNA, especially for these cases.

Compared with radiologists, the AI demonstrated superior ability in identifying benign nodules initially assessed as being at malignant risk, surpassing the median performance of both senior and junior radiologists. Senior and junior radiologists were categorized by years of practice rather than absolute expertise. While senior radiologists had a higher correct classification rate than the juniors, the internal dispersion remained large, with most still falling below the AI’s level and only 2 approaching or exceeding its performance. Cultivating senior radiologists is exceedingly challenging and takes a long time, whereas AI effectively compensates for the shortage of senior radiologists.

This underscores AI’s significant potential for clinical application, reducing unnecessary FNAs and optimizing nodule management. It saves both human resources and time while ensuring high accuracy and minimizing missed diagnoses. Integrating AI into clinical practice ensures more consistent identification, reduces unnecessary health care costs, eases the burden on the health care system, and saves time and effort for radiologists.

From another perspective, AI re-evaluation resulted in 123 erroneous identifications (incorrectly labeling them as benign) among 4572 (2.7%) nodules. These malignant nodules represent a risk in AI re-evaluation. Balancing the risks and benefits of AI re-evaluation is crucial. Fortunately, our subgroup analysis revealed that all of these errors were papillary thyroid carcinomas, with 70 out of 123 (56.9%) being papillary thyroid microcarcinomas. Although the error classification rate was significantly lower than the correct classification rate and papillary thyroid cancer, especially microcarcinoma, is generally indolent with low mortality, we recommend active surveillance for these nodules. Given the error classification rate in intermediate- and high-suspicion nodules (28/165, 17% and 18/90, 20%, respectively), intensified surveillance is clinically warranted for these cohorts. Conversely, low-suspicion nodules demonstrated significantly higher diagnostic reliability (77/2587, 3%), justifying extended surveillance intervals.

This study has several limitations. First, the data were obtained exclusively from 3 centers in China, limiting the generalizability of the findings. We anticipate that expanding the datasets to include real-world data from other regions will enable a more comprehensive validation of the AI model’s performance. Second, the AI system we tested was limited to analyzing grayscale images, leaving valuable information such as clinical data and color Doppler features insufficiently evaluated. Third, since this study reflects the application of AI in real clinical settings, there is a potential for bias introduced by biopsy decisions influenced by clinical judgment or patient preferences. Fourth, no prospective studies have been conducted to further validate the AI’s application and its impact in clinical practice.

### Conclusions

In conclusion, we conducted a retrospective study to evaluate the potential of AI in clinical practice for identifying benign nodules from those initially classified as at malignant risk by radiologists. Our findings demonstrated that incorporating AI in the pre-FNA stage can significantly reduce the number of unnecessary FNAs, optimize the management of thyroid nodules, and outperform the average diagnostic accuracy of both junior and senior radiologists. Thus, including AI in the management of thyroid nodules may be a new and promising exploration to reduce the rate of unnecessary FNAs. To fully explore the potential of AI in thyroid nodule diagnosis, continued collaboration among AI developers, radiologists, and clinicians is essential.

## Supplementary material

10.2196/71740 Multimedia Appendix 1 Ultrasound equipment used in datasets 1 and 2.

10.2196/71740 Multimedia Appendix 2 The ratio of correct classification rate and error classification rate according to different suspicion categories in dataset 1.

10.2196/71740 Multimedia Appendix 3 The comparison between radiologists and AI in dataset 2. Junior radiologists with less than 10 years’ experience. Senior Radiologist with 10 years or more than 10 years of experience. The red dotted line in the figure S2a represents the successful identification rate of AI, which is 81.4%. The red dotted line in Figure S2b represents the correct classification rate of AI of 82.8%.

## Abbreviations

AI: artificial intelligence
ATA: American Thyroid Association
AUC: area under the curve
FNA: fine needle aspiration
