# Supplementary material for: Optimizing Thyroid Nodule Management With Artificial Intelligence: Multicenter Retrospective Study on Reducing Unnecessary Fine Needle Aspirations
Source: JMIR Med Inform. 2025 Jul 30;13:e71740. doi: 10.2196/71740 (PMC12310072; doi:10.2196/71740)
Supplement: Multimedia Appendix 1 [file medinform-v13-e71740-s001.docx]

**Multimedia Appendix 1**

Supplementary table 1: Ultrasound equipment used in Dataset 1 and Dataset 2 .

| Hospital | Machine | Manufacturer |
| --- | --- | --- |
| Shanghai Tenth People’s Hospital | Aixplorer | Supersonic Imagine |
|  | Philips (IU22, EPIQ 7) | Philips Medical Systems |
|  | GE (Logiq E9, Logiq S8) | GE Medical Systems |
|  | Mindray Resona (7T, DC-8) | Mindray Medical International |
|  | Canon Aplio (i800) | [Canon Medical Systems](http://www.baidu.com/link?url=CxKTPm06KgxzUGhMwf5W2iDThi50pP4iNxlRNjpDpxKfyAfkdW9BBnFkb-AX1cuTLZ3YklxhZJVG70EFwmRtVLt7IAhM6KCup8qEMoj4yvS) |
| Sichuan Provincial People's Hospital | GE (Logiq E9) | GE Medical Systems |
|  | Canon Aplio (i800) | [Canon Medical Systems](http://www.baidu.com/link?url=CxKTPm06KgxzUGhMwf5W2iDThi50pP4iNxlRNjpDpxKfyAfkdW9BBnFkb-AX1cuTLZ3YklxhZJVG70EFwmRtVLt7IAhM6KCup8qEMoj4yvS) |
| Zhongshan Hospital | GE (Logiq E10s) | GE Medical Systems |
|  | Philips (HD15) | Philips Medical Systems |
|  | Mindray Resona (7T, DC-8) | Mindray Medical International |
