# Supplementary material for: Optimizing Thyroid Nodule Management With Artificial Intelligence: Multicenter Retrospective Study on Reducing Unnecessary Fine Needle Aspirations
Source: JMIR Med Inform. 2025 Jul 30;13:e71740. doi: 10.2196/71740 (PMC12310072; doi:10.2196/71740)
Supplement: Multimedia Appendix 2 [file medinform-v13-e71740-s002.docx]

**Multimedia Appendix 2**

**
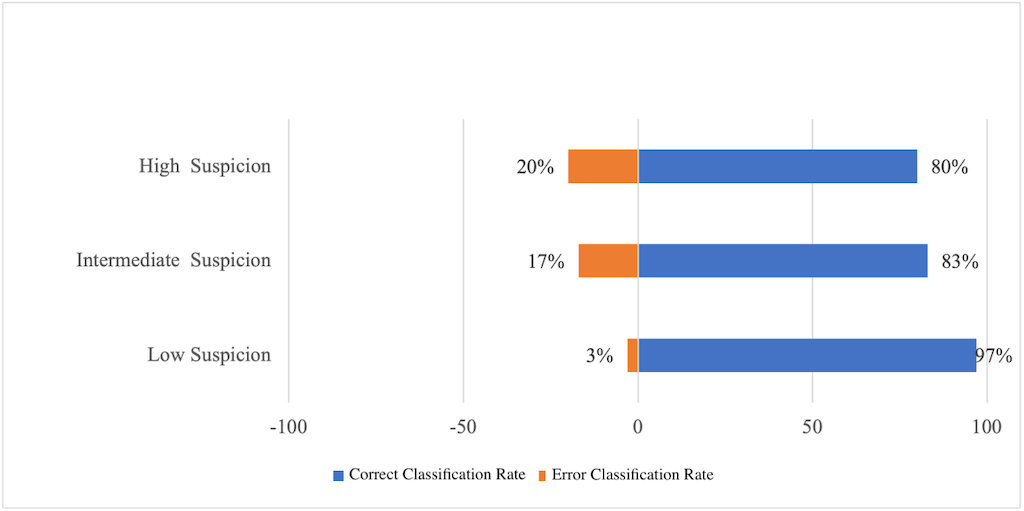
**

**Supplementary Figure 1: The ratio of Correct Classification Rate and Error Classification Rate according to different suspicion category in Dataset 1.**
