# Supplementary material for: Optimizing Thyroid Nodule Management With Artificial Intelligence: Multicenter Retrospective Study on Reducing Unnecessary Fine Needle Aspirations
Source: JMIR Med Inform. 2025 Jul 30;13:e71740. doi: 10.2196/71740 (PMC12310072; doi:10.2196/71740)
Supplement: Multimedia Appendix 3 [file medinform-v13-e71740-s003.docx]

**Multimedia Appendix 3**

**
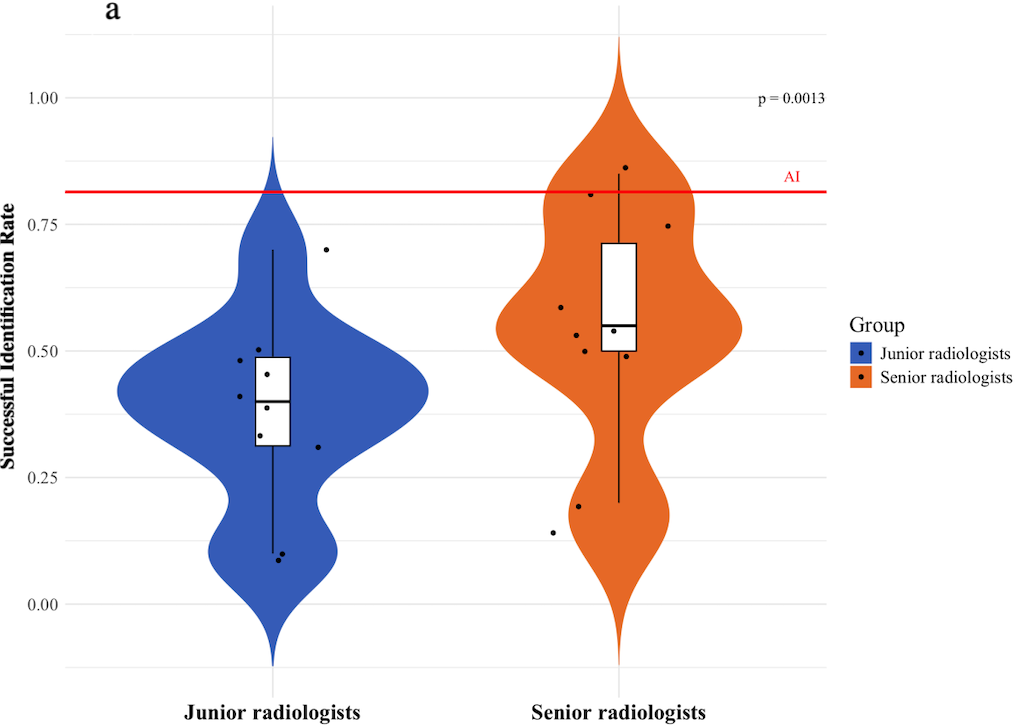
**

**
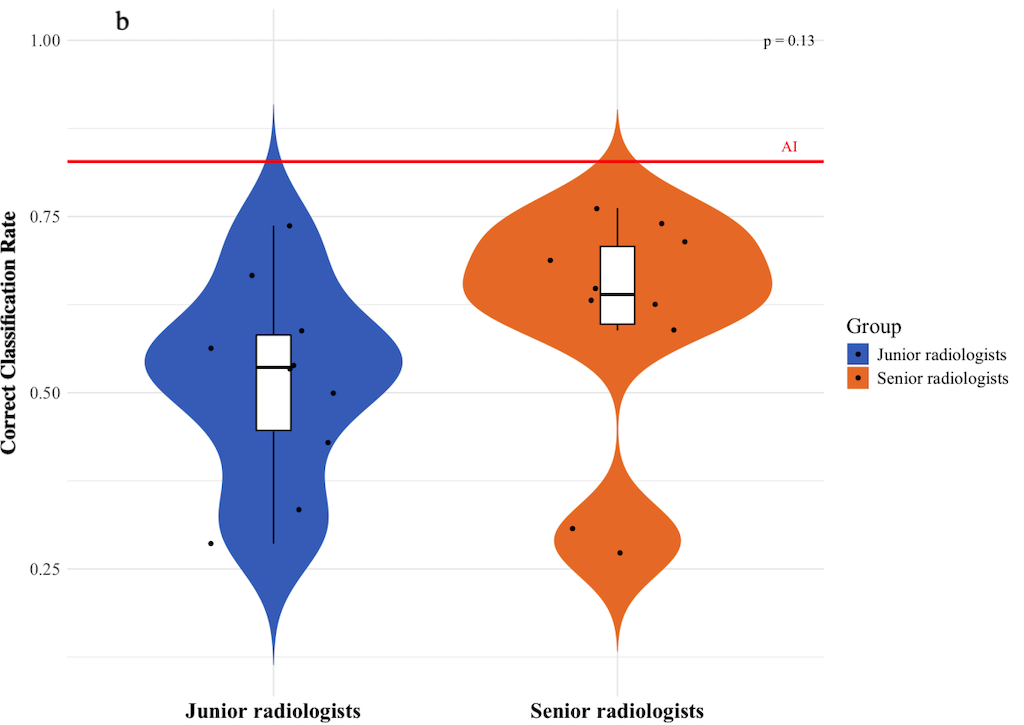
**

**Supplementary Figure 2: The comparison between radiologists and AI in Dataset 2.**

Junior radiologists with less than 10 years' experience.

Senior Radiologist with 10 years or more than 10 years' experience.

The red dotted line in in the figure S2a represents the Successful Identification Rate of AI is 81.4%.

The red dotted line in the figure S2b represents the Correct Classification Rate of AI is 82.8%.
